# The Crystal Structure of the N-Terminal Region of BUB1 Provides Insight into the Mechanism of BUB1 Recruitment to Kinetochores

**DOI:** 10.1016/j.str.2008.10.015

**Published:** 2009-01-14

**Authors:** Victor M. Bolanos-Garcia, Tomomi Kiyomitsu, Sheena D'Arcy, Dimitri Y. Chirgadze, J. Günter Grossmann, Dijana Matak-Vinkovic, Ashok R. Venkitaraman, Mitsuhiro Yanagida, Carol V. Robinson, Tom L. Blundell

**Affiliations:** 1 Department of Biochemistry, University of Cambridge, Cambridge CB2 1GA, UK; 2 CREST Research Program JST, Graduate School of Biostudies, Kyoto University, Kyoto 606-8501, Japan; 3 Molecular Biophysics Group, School of Biological Sciences, University of Liverpool, Liverpool L69 7ZB, UK; 4 Department of Chemistry, University of Cambridge, Cambridge, UK; 5 Hutchison/MRC Research Centre, Cambridge CB2 0XZ, UK

**Keywords:** PROTEINS, CELLBIO

## Abstract

The interaction of the central mitotic checkpoint component BUB1 with the mitotic kinetochore protein Blinkin is required for the kinetochore localization and function of BUB1 in the mitotic spindle assembly checkpoint, the regulatory mechanism of the cell cycle that ensures the even distribution of chromosomes during the transition from metaphase to anaphase. Here, we report the 1.74 Å resolution crystal structure of the N-terminal region of BUB1. The structure is organized as a tandem arrangement of three divergent units of the tetratricopeptide motif. Functional assays in vivo of native and site-specific mutants identify the residues of human BUB1 important for the interaction with Blinkin and define one region of potential therapeutic interest. The structure provides insight into the molecular basis of Blinkin-specific recognition by BUB1 and, on a broader perspective, of the mechanism that mediates kinetochore localization of BUB1 in checkpoint-activated cells.

## Introduction

The missegregation of sister chromatids during mitosis results in the loss or gain of chromosomes in daughter cells (aneuploidy). This disastrous outcome is avoided by the mitotic checkpoint for spindle assembly (SAC), which monitors the proper assembly of the mitotic spindle and blocks the onset of anaphase until the kinetochores of all chromosomes receive a bipolar attachment to spindle microtubules. BUB1 and BUBR1 (BUB1-related kinase, known as MAD3 in yeast) are multidomain proteins that play central roles in this process, working together with other kinetochore-bound components including MAD2, BUB3, CDC20, and MPS1.

**Figure 1:**
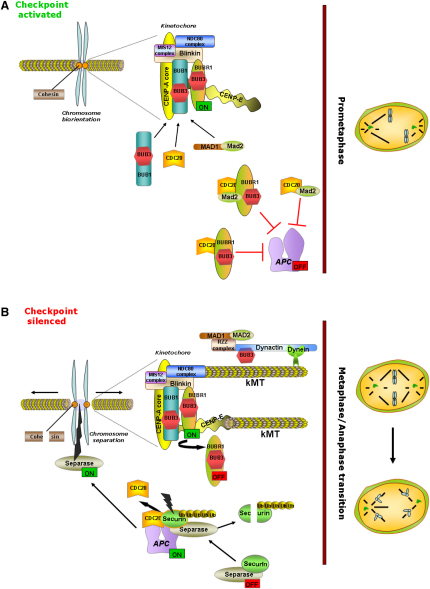
Model Showing the Functions of BUB1 and BUBR1 in the Mitotic Spindle Checkpoint during Mitotic Progression (A) In prometaphase, the nuclear envelope breaks down and microtubules emanating from opposite poles attach to the kinetochores of individual sister chromatids. Core checkpoint components BUB1, BUBR1, BUB3, MAD1, and MAD2 are recruited to unattached kinetochores in SAC-activated cells. Kinetochore localization of BUB1 and BUBR1 is mediated by the mitotic kinetochore protein Blinkin. The latter also establishes physical interaction with the MIS12 and NDC80 complexes. Cytosolic BUBR1, BUB3, MAD2, and CDC20 associate to form mitotic checkpoint complexes, which interact with the anaphase promoter complex/cyclosome (APC/C) to render it inactive. (B) In metaphase, the bipolar attachment and alignment of all chromosomes at the center of the cell is reached and APC/C-CDC20 inhibition released by silencing of the SAC. This is followed by the onset of anaphase, in which sister chromatids separate and are pulled toward opposite poles of the cell. When the checkpoint is silenced, securing can be ubiquitinated by APC/C and degraded. This results in the release and activation of separase, which leads to the cleavage of mitotic cohesions at centromeres and chromosome arms to cause chromosome separation and mitotic progression from M-phase to interphase.

Checkpoint proteins are not recruited simultaneously to kinetochores. Instead, they obey a temporal order of assembly where the recruitment of the later proteins is dependent on the prior recruitment of early ones (Sharp-Baker and Chen, 2001; Vanoosthuyse and Hardwick, 2005; Wong and Fang, 2006). BUB1 is recruited very early in prophase (Wong and Fang, 2006), promotes the assembly on centromeres of components of the outer kinetochore (such as BUBR1 and CENP-F), and is essential for assembly of the functional inner centromere (Taylor et al., 1998; Boyarchuk et al., 2007). It accumulates at the kinetochore in SAC-activated cells and assures the correct kinetochore formation (revised in Logarinho and Bousbaa, 2008). BUBR1, by contrast, functions as a downstream component that is recruited to the kinetochore at a later stage. Kinetochores that are not correctly attached to microtubules recruit components of the mitotic checkpoint, initiating a signaling cascade that results in CDC20-dependent inhibition of the anaphase-promoting complex or cyclosome (APC/C) (Figure 1). In *Drosophila*, loss of BUB1 causes chromosome missegregation and lethality (Basu et al., 1999), whereas in mice BUBR1 insufficiency causes infertility and early aging (Baker et al., 2004). In human cells, defects in the mitotic checkpoint proteins BUB1 and BUBR1 have been associated with various classes of cancer (Cahill et al., 1998; Hanks et al., 2004; Kops et al., 2005).

Sequence comparison of BUB1 and BUBR1 shows that these proteins share a common architecture: a conserved N-terminal region, a central nonconserved region that contains the binding region for other mitotic checkpoint components such as BUB3, and a C-terminal serine/threonine kinase domain. The N-terminal region mediates the binding of Hs-BUB1 to the mitotic kinetochore protein Blinkin (a protein also commonly referred to as AF15q14); the interaction is essential for the kinetochore localization of this protein and its function in cell cycle arrest induced by SAC activation (Kiyomitsu et al., 2007). Residues 1–179 of fission yeast BUB1 are necessary for targeting the protein Shugoshin 1 (SGO1) to centromeres (Vaur et al., 2005). Other reports have shown that deletion of the N-terminal residues 28–160 of Sp-BUB1 results in a truncated protein unable to recruit BUB3 and MAD3 to kinetochores (Vanoosthuyse et al., 2004). Furthermore, it has been shown that deletion of residues 1–47 of Hs-BUBR1 severely impairs mitotic checkpoint activity (Harris et al., 2005), and the N-terminal region of yeast MAD3 mediates the binding of CDC20 (King et al., 2007).

Using an approach that combines bioinformatics with biochemical and biophysical methods, we have mapped the boundaries of the conserved N-terminal region of BUB1 and BUBR1 (MAD3) proteins (Bolanos-Garcia et al., 2005; Beaufils et al., 2008). In human BUB1, it encompasses residues 1–200, whereas that of budding yeast spans the first 230 residues. We now describe the 1.74 Å resolution crystal structure of the conserved N-terminal region of Sc-BUB1 and relate the structure to the binding of Hs-BUB1 to the mitotic checkpoint factor Blinkin, an interaction that is essential for the recruitment of Hs-BUB1 and Hs-BUBR1 (MAD3) to the kinetochore.

## Results

### Deletion of Residues 1–28 Does Not Disrupt Domain Stability

**Figure 2:**
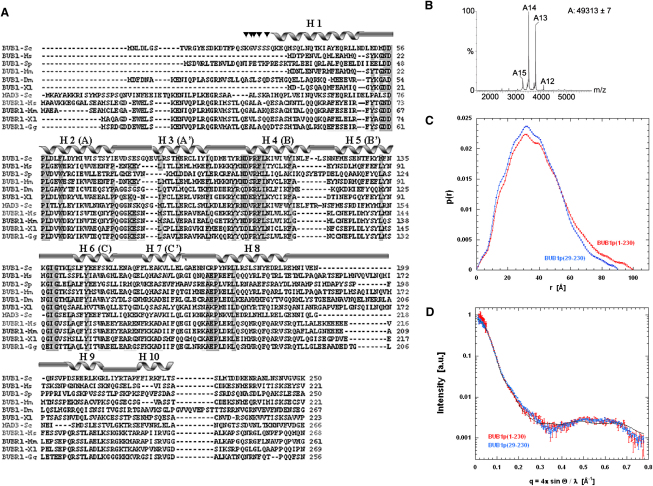
Sequence Conservation of the N-Terminal Domain of BUB1 and BUBR1 (A) The ten α helices seen in the structure of Sc-BUB1_(29-230)_ are indicated above the alignment by solid ribbons. Fully conserved residues in BUB1 and BUBR1 that are located in loop regions are highlighted in dark gray. Sc, *Saccharomyces cerevisiae*; Hs, *Homo sapiens*; Sp, *Schizosaccharomyces pombe*; Mm, *Mus musculus*; Dm, *Drosophila melanogaster*; Xl, *Xenopus laevis*; Gg, *Gallus gallus*. Six representative BUB1 sequences and five of BUBR1 were aligned using ClustalW (Thompson et al., 1994). The inverted triangles above the alignment (▾) show the trypsin cleavage sites identified after limited proteolysis and proteomics analysis. (B) Nano-ES mass spectrum of 45 μM sample (+15 to +12), in the m/z range 3000 and 4500. A single charge state distribution was observed centered on charge +14 at m/z 3510. These values correspond to a molecular weight of 49,313 Da, consistent with a dimer (Mw of monomeric and dimeric Sc-BUB1_[29-230]_ calculated from the amino acid sequence is 24,226 Da and 48,452 Da, respectively). (C) Comparison of the distance distribution [p(r)] function of Sc-BUB1_(29-230)_ (blue) against that of Sc-BUB1_(1-230)_ (red) provides evidence for conformational heterogeneity due to structural flexibility of the region encompassing residues 1–28. (D) The SAXS scattering profile simulation (continuous lines) based on the dimer observed in the crystal structure demonstrates that the overall conformation of Sc-BUB1_(1-230)_ (red) and Sc-BUB1_(29-230)_ (blue) is essentially conserved in solution.

Although the N-terminal region of BUB1 from *Saccharomyces cerevisia*e comprising residues 1–230 (Sc-BUB1_[1-230]_) was successfully overexpressed in *Escherichia coli* as a soluble protein and purified to homogeneity, numerous attempts to crystallize it were unsuccessful. To define a stable domain suitable for structural studies, we first investigated the role of residues at the N terminus. Secondary structure prediction programs consistently predicted a flexible region (residues 23–28) between the first putative α helix of the N-terminal region of BUB1 (predicted to encompass residues 9–22) and the second (H1 in our crystal structure, residues 32–49). To test this hypothesis, Sc-BUB1_(1-230)_ was subjected to limited proteolysis coupled to mass spectrometry and Edman degradation analysis. The larger stable species had their N termini at residues 24, 26, 27, or 28 (Figure 2A).

Nano-electrospray (nano-ES) mass spectra, analytical gel filtration, and solution X-ray scattering (SAXS) (Figure 2B; see Supplemental Data available online) show that this region forms stable dimers in aqueous solutions. We used SAXS to determine the compactness and the maximum extension of Sc-BUB1_(1-230)_ and the shorter construct Sc-BUB1_(29-230)_ in aqueous solutions. A scattering profile simulation using the atomic structure of the dimer and its comparison with the experimental scattering data produces a goodness-of-fit value of chi of 8.8 for Sc-BUB1_(1-230)_ and 5.6 for Sc-BUB1_(29-230)_ (Figure 2C). This confirms the good agreement between the structure of (Sc-BUB1_[29-230]_) in solution and the crystalline state. Moreover, the comparison of the SAXS scattering profile of Sc-BUB1_(1-230)_ and Sc-BUB1_(29-230)_ shows that the radius of gyration of Sc-BUB1_(1-230)_ decreases from 30.4 Å to 28.4 Å and the maximum particle size narrows from 100 Å to 90 Å when the N-terminal 28 residues are removed (Figure 2D). Hence, both hydrodynamic parameters are consistent with an N terminus extended away from the main part of the structure of Sc-BUB1_(1-230)_. Based on these analyses, the BUB1 construct encompassing residues 29–230 (Sc-BUB1_[29-230]_) was cloned and overexpressed in *E. coli*.

### Sc-BUB1_(29-230)_ Has a Tetratricopeptide-like Fold

**Figure 3:**
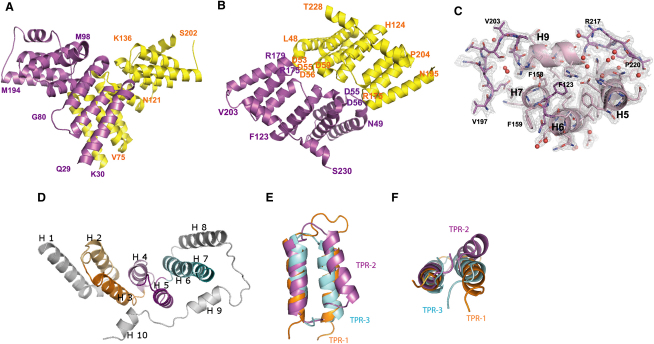
Overall Structure of the N-Terminal Domain of BUB1 (A) The two molecules observed in the crystal asymmetric unit associate to form a dimer with noncrystallographic two-fold symmetry. (B) The structure viewed 90° rotated along the minor axis. (C) Electron density for α helices H5, H6, H7, and H9 and the connecting loop (after density modification) contoured at 1.4 σ. The final, refined model is shown using a ball-and-stick representation. The α helices, loops, and side chains are clearly visible in the initial map. Water molecules are shown as red spheres. (D) Ribbon diagram showing that this domain consists of ten α helices with a core arrangement of a triple repeat of the TPR motif (TPR1 orange; TPR2 magenta; TPR3 cyan). (E,F) Superposition of the three TPRs of Sc-BUB1_(29-230)_. Each molecule representation was generated with Pymol (DeLano, 2002).

The crystal structure of the N-terminal region of Sc-BUB1_(29-230)_ from budding yeast was solved at 1.74 Å resolution (Table 1 and Figures 3A–3C), and contains two molecules of BUB1 in the asymmetric unit, related by a noncrystallographic two-fold axis, forming a homodimer. The two protomers are associated primarily by hydrogen bonds and van der Waals interactions and involve clusters of conserved residues E52-P57, M98-K109, and R175-R182. Salt bridges between the oxygen atom of residue D53 of one protomer and the nitrogen atoms (NH1, NH2) of R179 and R182 of the other, as well as between D55 of one protomer and K109, R175, and R179 of the other, contribute further to the stability of the homodimer. The nature of these interactions gives confidence that this represents the dimer observed in solution. The average surface area of the homodimer interface measured as the change in the solvent accessible surface area, ΔASA, is 965 Å^2^, which accounts for 8.6% of the total surface area of one Sc-BUB1_(29-230)_ protomer. Although small, this ΔASA value is within the range observed in many obliged dimers (i.e., ΔASA ≥ 800 Å^2^; Jones and Thornton, 1996).

The structure of the Sc-BUB1_(29-230)_ protomer consists of ten α helices (H1–H10) that contribute three major features: a single α helix (H1), a tandem repeat of three units of the tetratricopeptide (TPR) motif (H2-H7), and a C-terminal region encompassing three α helices (H8-H10) (Figure 3D). Each TPR unit (TPR1, TPR2, and TPR3, numbered from the N terminal) comprises two antiparallel α helices, arranged as a helix-turn-helix hairpin. Superposition of TPR units of Sc-BUB1_(29-230)_ shows that they adopt similar conformations despite the low sequence identity (Figures 3E and 3F). TPR1 and TPR3 show low sequence identity (4%) but have a 1.81 Å root-mean-square deviation (rmsd) of Cα atoms. TPR2 and TPR3 have sequence identity of 8.5% and 2.7 Å rmsd of Cα, and TPR1 and TPR2 share a sequence identity of 5.7% and 2.8 Å rmsd of Cα. The linker regions between TPR units differ in length and local structure: TPR1 and TPR2 are connected by a 3_10_ helix (residues E99-R102) linking helices H3 and H4, whereas TPR2 and TPR3 are connected by a short loop (residues I138-K141) between helices H5 and H6.

Although there is no position characterized by an evolutionary invariant residue, a sequence pattern of small and large hydrophobic residues in the TPR units can be identified in Sc-BUB1_(29-230)_. Small hydrophobic residues are mainly at the positions of closest contact between the α helices that define a TPR unit, whereas large hydrophobic residues (predominantly phenylalanine and isoleucine) form the interfaces between adjacent TPRs. Hydrophobic residues are essential for the structural integrity of many TPRs (Main et al., 2003), so it is expected that those located at equivalent positions in Sc-BUB1_(29-230)_ play a similar structural role. The fact that residues identified as well conserved in many TPRs (D'Andrea and Regan, 2003) are poorly conserved in Sc-BUB1_(29-230)_ (and also in BUB1/BUBR1 from different species) indicates that important deviations from the canonical 34-residue TPR motif are tolerated in these proteins. However, some features typical of other TPR motifs can be recognized in Sc-BUB1_(29-230)_. For example, the three TPR units assemble into a relatively extended structure to form a regular series of antiparallel α helices rotated relative to one another by a constant 24°. The uniform arrangement of neighboring α helices gives rise to the formation of a right-handed superhelical structure with a continuous concave surface on one side and a contrasting convex surface on the other. Many of the features that appear to stabilize the fold are conserved in the close homologs. For example, the fully conserved residues D56 and R106 form a salt bridge, which favors the interaction between helices H2 and H4 and stabilizes the end points of the intervening, extended loop. R106 also contacts the fully conserved residue D104 (H4), thus allowing a close packing of helices H3 and H4.

The amphipathic helix 8, which encompasses residues Y177 to M194, has the features of a “capping helix.” Such capping helices have been identified in the TPR motifs of PP5 and HOP (Das et al., 1998; Scheufler et al., 2000). In structures that consist of more than one TPR motif, the C-terminal non-TPR component can adopt different kinds of conformations: it can be unstructured, it can bind to the concave face as an extended polypeptide, or it can assume a completely independent domain organization. Beyond H8, the C-terminal 30 residues of Sc-BUB1_(29-230)_ are extended and include two short, α helices (H9 residues S206 to L214 and H10 residues F221 to T228), linked by a short irregular region (residues I215 to P220). Helices H9 and H10 lie parallel to the long axis of the TPR core, establishing extensive polar contacts with TPR helices H3, H4, H5, H6, and H7. These interactions involve ion pairs E127-R213, E161-R209, K163-E198, and E171-R207.

Both short-range and long-range interactions are essential for the stability of tandem arrays of the TPR motif (Main et al., 2005). The extent of these classes of interactions between α helices of the TPR motifs of BUB1 might explain the documented instability of deletion mutants of the N-terminal regions of Hs-BUBR1 (Bolanos-Garcia et al., 2005 and this report) and Sc-MAD3 (Larsen et al., 2007). Of interest in this context is the observation that thermal unfolding of Sc-BUB1_(29-230)_ measured by far-UV circular dichroism (CD) is highly cooperative (Figure S1A and Supplemental Data). It follows a two-state transition with a Tm of 63°C and ΔH of 42 Kcal/mol, a value that is within the range commonly observed in other TPR domains of similar size (i.e., 40–65 Kcal/mol). Although dimeric Sc-BUB1_(29-230)_ retains the folded state in the pH range of 7 to 10 as shown by far-UV CD, it is predominantly disordered at pH ≤ 5 (Figure S1B and Supplemental Data). Interestingly, the pH stability profile of Sc-BUB1_(29-230)_ is comparable to that of the equivalent region in Hs-BUBR1, residues 1–204 (Bolanos-Garcia et al., 2005).

**Figure 4:**
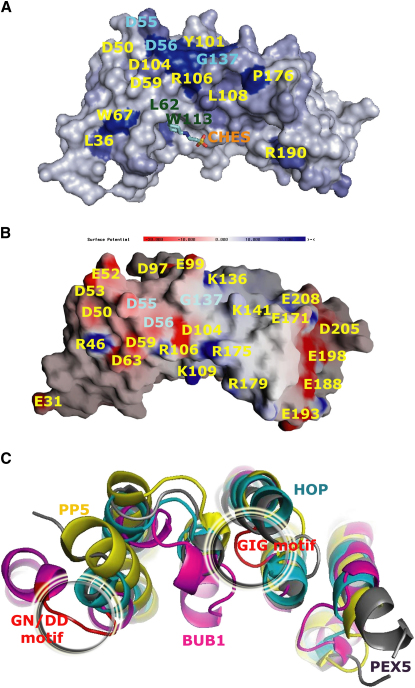
Projection of Conserved Regions onto the Protein Surface (A) Residue conservation is shown according to the sequence-conservation score of ProSkin (Deprez et al., 2005). Blue and white denote conserved and variable regions, respectively. Conserved residues of the GN/DD and GIG motifs are shown in blue, two of the residues that define the hydrophobic pocket are shown in green, and the CHES molecule is shown using a ball-stick representation. (B) Electrostatic surface potential computed with GRASP (Nicholls et al., 1991). The electrostatic potential is contoured at the 10 kT/e level, with red denoting negative potential and blue denoting positive potential. Note the prominent acidic loop defined by the conserved residues E52 to D56 and that defined by the less conserved residues E167, E171, E188, E193, E198, D205, and E208. The structure is view rotated 90° along the vertical axis from that in (A). (C) The loops encompassing residues D53-D56 and G137-I-G139 (red) show conformations unique among TPRs of high local-structure similarity (Hs-PEX TPR is gray, Hs-HOP TPR2 is cyan, and Hs-PP5 TPR is yellow).

The pattern of sequence conservation of Sc-BUB1_(29-230)_ (Figure 4A) shows that loop residues D56, Y101, D104, G137, I138, G139, and P176 are fully conserved in BUB1 and BUBR1 across species. These residues define a continuous surface area that is extended by the conservation of residues D55 and N103. Charged residues are clustered in two regions (Figure 4B), the most prominent of which is acidic, defined by residues D50, E52, D53, D55, D56, D59, D63, D97, E99, and D104 and involving helices H2 and H4. A second cluster is defined by the basic residues R106, K109, K141, R175, R179, and R182 (H4 and H8). As shown in Figures 3A and 3B, some of the conserved charged residues of this region are involved in dimer formation.

### Structural Basis for the Interaction of BUB1 with Blinkin

**Figure 5:**
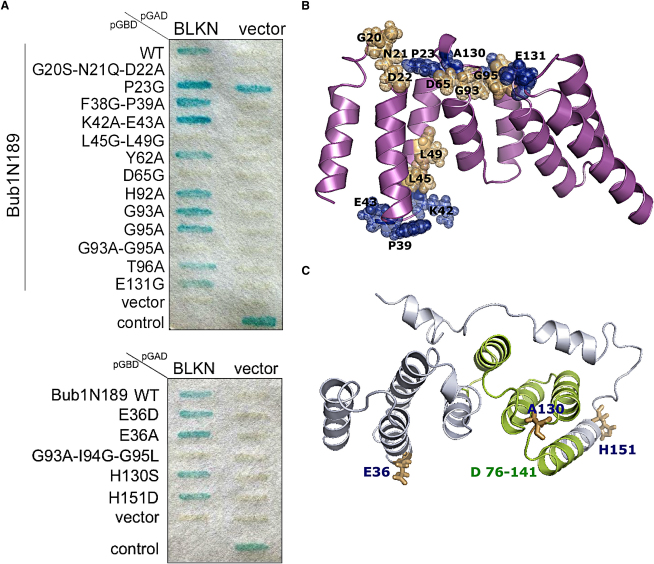
Analysis of the Hs-BUB1-Blinkin Interaction (A) Yeast two-hybrid analysis of diverse Hs-BUB1 mutants. The effect of the mutant P23G could not be established as it showed self-activation. (B) Projection of residues important for binding Blinkin onto the protein surface (salmon color). Residues whose mutation did not compromise the binding with Blinkin are shown in blue. (C) Mapping of Hs-BUB1 mutations associated with cancer. Residues absent in the deletion mutant Δ76–141 are shown in green and residues E36D, A130S, and H151D in brown.

Hs-Blinkin (BUB-linking kinetochore protein) is a large (265 kDa), predominantly disordered protein that constitutes the kinetochore target of BUB1 and BUBR1 in human cells. Although the boundaries of the N-terminal domain of Hs-BUB1 that physically interacts with Blinkin were ill-defined due to the lack of direct structure information at the time, the study shows that the N-terminal regions of Hs-BUB1 (residues 1–150) and Hs-Blinkin (1–728) are involved in the interaction (Kiyomitsu et al., 2007). The high conservation of residues (≈60% similarity between Sc-BUB1_[29-230]_ and its human counterpart) indicates they are likely to have similar structures. Indeed, the combined use of the structure information with functional analyses in vivo allows us to define the Blinkin binding region of Hs-BUB1. In the later, the substitution of A106 by D or W, the insertion of G between A104 and W105 and the substitution of L122 by G all disrupted the interaction of this protein with Blinkin (Kiyomitsu et al., 2007). The analysis of the crystal structure of Sc-BUB1_(29-230)_ strongly suggests that the impaired interaction between these Hs-BUB1 mutants and Blinkin is due to the disruption of stabilizing interactions among α helices of the TPR units. It also allows the identification of two highly conserved motifs (GN/DD and GIG) that, as explained below, play a key role in the binding of Hs-BUB1 to Blinkin. Superposition of the crystal structure of Sc-BUB1 with those of proteins containing similar TPRs (i.e., Hs-PEX TPR, Hs-HOP TPR2, and Hs-PP5 TPR) shows the GN/DD and GIG motifs of Sc-BUB1 exhibit unique conformations (Figure 4C). Mutation of residues in each motif (i.e., G20-D22 and G93-G95) to alanines totally abolished the interaction of Hs-BUB1 with Blinkin (Figure 5A). In contrast, mutation of other highly conserved loop residues (F38A-P39A; K42A-E43A) had no effect on the Hs-BUB1-Blinkin interaction (Figure 5A). The observation that the mutation D73K in Hs-BUBR1 (which was wrongly numbered as D67 in Harris et al., 2005 and is equivalent to D56 in Sc-BUB1) results in a defective mitotic checkpoint protein confirms the important role of the GN/DD motif for the interaction between Hs-BUB1 and Blinkin.

### The GIG Motif Is Essential for SAC Function

The GIG motif (G_137_I_138_G_139_) of budding yeast BUB1 is fully conserved in the BUB protein family and across species. It has been suggested that replacement of residues that define the GIG motif by alanines (also wrongly numbered as residues G140-I141-G142 in Harris et al., 2005) in Hs-BUBR1 results in more than 50% reduction in the SAC function by the expression of these mutants. Further experiments conducted in a strain containing only the mutant form of Sc-MAD3, G156A-S159A, show that the mutant protein was expressed at wild-type levels but abolished the interaction of Sc-MAD3 with Sc-CDC20 (Hardwick et al., 2000). Consistent with these observations, circular dichroism and analytical gel filtration experiments showed the double Sc-BUB1_(29-230)_ mutant G137A-G139A is predominantly dimeric, and exhibits comparable thermal stability and a native-like fold (Figure S1C). The crystal structure of Sc-BUB1_(29-230)_ shows that the second and third residues of the GIG motif are buried. It also shows that the two glycine residues exhibit a positive phi torsion angle. Interestingly, we observed that single mutations of the GIG motif in Hs-BUB1 (i.e., G93A and G95A) and of residues flanking the GIG motif (H92A and T96A) did not affect the interaction of Hs-BUB1 with Blinkin, whereas the double mutant G93A-G95A and the triple mutant G93A-I94G-G95A both abolished Blinkin binding (Figure 5A). Given the well-defined structural roles of the residues of the GIG motif, it is probable that structural changes become increasingly delocalized, the greater the difference in size of the substituted residues from the wild-type and the greater the number of substituted residues. Structural changes that result from increased delocalization might provide an explanation of the impaired interaction of the Sc-MAD3 G156A-S159A double mutant with Sc-CDC20 (Hardwick et al., 2000). Interestingly, the GIG motif of Hs-BUBR1 seems to play a similar important role in binding Blinkin because several mutants of this motif disrupt the interaction (S.D., V.M.B-G., and T.L.B., unpublished data). When mapped onto the surface, the Blinkin binding region of Hs-BUB1 defines a discontinues area involving the loop connecting helices H1-H2, residues in H3 that form part of one small hydrophobic pocket as well as the loops linking the short helix 3_10_-H4 and H5-H6 (Figure 5B).

### Cancer-Associated Mutations Do Not Disrupt the Interaction with Blinkin

When the mutations that have been identified and associated with aneuploidy and cancer progression (Table 2) are mapped onto the surface, their impact on the structure can be assessed. The Hs-BUB1 mutations E36D, A130S, and H151D lie in regions that connect the α helices of the TPR units (Figure 5C), whereas deletion of helices H5 to H7 and a half of H8 in the mutant Δ76-141 could not be accommodated without considerable disturbance of the entire domain structure (Figure 5C). These qualitative predictions are supported by SDM (Topham et al., 1997), I Mutant (Capriotti et al., 2005), and other computer programs that predict stability changes caused by punctual mutations (Table 2). Interestingly, none of these mutations were predicted to lead to great reductions in stability, in good agreement with the retention of a SAC response of these mutants in vivo (Tighe et al., 2001).

APC is a conserved 1.5–1.7 MDa asymmetrical complex that ubiquitinates a multitude of proteins. APC is composed of at least 11 subunits, most of which are evolutionarily conserved including the cell cycle proteins CDC16 and CDC23. Mutations within TPRs of CDC23 (Sikorski et al., 1990) and the CDC16 homolog CUT9 (Samejima and Yanagida, 1994) cause mitotic arrest at the metaphase-to-anaphase transition, probably due to the incorrect packing of neighboring α helices (Sikorski et al., 1990). In a similar fashion, mapping of Hs-BUB1 mutations onto Sc-BUB1_(29-230)_ shows some residues connecting α helices of the TPR units constitute a preferred location of mutations associated with genetic instability. It also suggests that the Hs-BUB1 mutations E36D, A130S, and H151D are likely to disrupt the packing and stability of TPR-forming α helices. Our functional studies in vivo suggest the Hs-BUB1 mutations E36D, A130S, and H151D, which contribute to chromosome instability in cancer cells, impaired the SAC through a mechanism that is not dependent of the direct interaction between Hs-BUB1 and Blinkin. One possibility is that cancer-associated mutations of the N-terminal domain of BUB1 impair stabilizing interactions with BUB1 residues C-terminal to this domain that are not present in the crystallized protein (for instance, with those located in the adjacent BUB3 binding region).

### Analysis of Protein-Protein Interaction Sites

**Figure 6:**
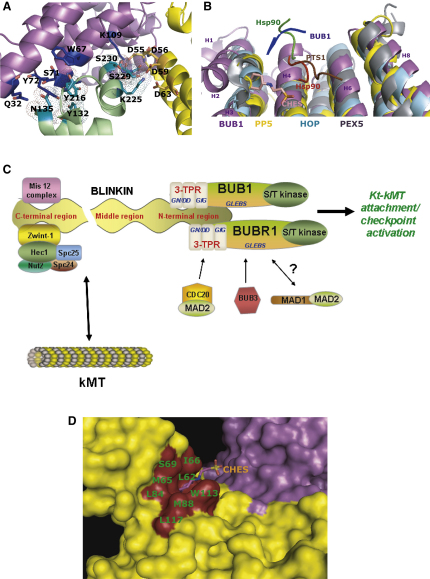
Mechanistic Implications (A) The two Sc-BUB1_(29-230)_ protomers (show in yellow and magenta, respectively) form a groove that binds the C-terminal residues I215-S230 of a symmetry-related molecule (green). Several of the conserved residues that define an acidic cluster (orange color) are shown in the yellow protomer. Residues of the other protomer (magenta) that interact with residues I215-S230 of a symmetry-related molecule are shown in blue. (B) Superposition of TPR-containing domains of Sc-BUB1, Hs-PP5, Hs-HOP, and Hs-PEX5 shows that they form a similar concave face. Hs-PP5 and Hs-HOP bind the same pentapeptide sequence MEEVD (Hsp90, blue and red, respectively) in a different mode. Peroxisomal targeting signal-1 peptide (PTS-1, brown) binds Hs-PEX5 in a manner that resembles that of Hsp90-HOP. In Hs-BUB1, residues of the Blinkin-binding region map onto α helices equivalent to those of Hs-PP5, Hs-HOP, and Hs-PEX5 engage in peptide binding, suggesting a similar binding mode. In Sc-BUB1_(29-230)_ various residues of these helices (H2 and H4) are involved in the interaction with one CHES molecule (salmon) and others with the C-terminal region of a symmetry-related Sc-BUB1 molecule (green), suggesting that other residues participate in additional protein-protein interactions. (C) The TPR-containing domain of Hs-BUB1 and Hs-BUBR1 bind to the N-terminal region of Blinkin, whereas the physical interaction of BUB3 with BUB1 and BUBR1 involves the GLEBS motif. Putative MAD1 and MAD2 binding sites can be identified in the region preceding the kinase domain. The different affinity of Blinkin for Hs-BUB1 and Hs-BUBR1 might permit the simultaneous control of the recruitment of these proteins to the kinetochore. (D) Close-up view of the interaction between CHES and the hydrophobic pocket of Sc-BUB1_(29-230)_. One CHES molecule is located inside the small hydrophobic pocket constituted by residues L62, M65, I66, S69, L84, M88, I110, W113, and L117 (brown area).

The analysis of the crystal structure combined with the use of bioinformatics tools suggests other residues might be involved in protein-protein interactions, supporting the notion that BUB1 acts as a molecular scaffold for the recruitment of other components of the SAC. In the crystal lattice, several conserved residues that are localized in a groove formed after dimerization show extensive interactions with the C-terminal residues I215-S230 of another Sc-BUB1_(29-230)_ protomer (Figure 6A). These interactions, which engage helices H1, H2 and H4 of one dimer and H10 of another, involve salt bridge formation between the Nz atom of K225 and the oxygen atoms of residues D59 and D63 as well as hydrogen bonding between residue pairs D55-S230, D56-S230, and D59-S229 (Figure 6A).

These interactions in Sc-BUB1_(29-230)_ resemble those seen in several TPR-peptide complexes such as the N-terminal TPR domains 1 and 2 of Hs-HOP with the C-terminal region of Hsc70 and Hsp90 respectively (Scheufler et al., 2000) and Hs-PEX5 with the peroxisomal targeting signal-1 (PTS1) (Gatto et al., 2000). A Dali search (http://www.ebi.ac.uk/dali/) for structural homologs returned parts of the TPR motif of Hs-protein phosphatase 5 (PP5) (Das et al., 1998) longer than 70 residues as the structure of highest local similarity with parts of Sc-BUB1_(29-230)_ (3.6 Å rmsd of Cα). The other hits were the TPR domains of Hs-Hsp70/Hs-Hsp90 organizing protein (HOP) (Scheufler et al., 2000) (3.6 Å rmsd of Cα) and Hs- receptor PEX5 (Gatto et al., 2000) (3.7 Å rmsd of Cα). Superposition of Sc-BUB1_(29-230)_ with the TPR-containing domain of Hs-PP5-Hsp90_(MEEVD),_ Hs-HOP-Hsp90_(MEEVD)_, and Hs-PEX5-PTS1 complexes shows that residues involved in peptide binding are located in equivalent α helices (Figure 6B), even though there is little sequence conservation of residues in equivalent positions in Sc-BUB1_(29-230)_.

Although the size of the Sc-BUB1_(29-230)_-I215-S230 interface (420 Å^2^) is rather small, it is comparable to those of the protein-peptide complexes Hs-HOP TPR1-Hsc70 and Hs-HOP TPR2-Hsp90 (430 Å^2^, 380 Å^2^, respectively) and higher than that of the Hs-PP5 TPR-Hsp90 complex (260 Å^2^). Even though the small sizes of the area buried would unlikely give tight binding, it can be anticipated that additional residues cooperate in BUB1-ligand interactions. We note that for instance the interaction of Hs-Hsp90 with full-length HOP does not rely on the Hs-HOP TPR2 binding site alone but also on other polypeptide segments of HOP. Yet the binding of Hsp90_(MEEVD)_ to Hs-HOP TPR2 seems to induce a conformational change that most likely generates further interaction areas within full-length HOP (Onuoha et al., 2008).

In all of the above TPR-peptide complexes, electrostatic interactions play an important stabilizing role. Use of the ODA method, a bioinformatics approach that facilitates the identification of potential interaction sites (Fernandez-Recio et al., 2004; Bolanos-Garcia et al., 2006), further supports this notion: the ODA method predicts that the Sc-BUB1_(29-230)_ region that extends toward the α helices H2, H4, and H6 is the most favorable for protein-protein interactions (Figure S2 and Supplemental Data). Furthermore, the residues of Sc-BUB1 shown to be engaged in protein dimerization in the crystal lattice and those involved in the interaction with C-terminal residues of another Sc-BUB1 molecule are located in positions equivalent to residues N21, D22 (GN/DD motif) and G93, I94, G95 (GIG motif) of Hs-BUB1, mutation of all of which affects binding Blinkin (Figure 5B).

Structure superposition of Hs-PP5, Hs-HOP, and Sc-BUB1 shows that residues N36, F39, and N67 of Hs-PP5 involved in binding Hsp90_(MEEVD)_ and N233, Y236, and N264 of Hs-HOP TPR2 involved in binding the same peptide Hsp90_(MEEVD),_ map onto the Sc-BUB1 2-(n-cyclohexylamino)ethane sulfonic acid (CHES)-binding residues L62, M65, and I110, respectively. Mutation of residues L45G and L49G in Hs-BUB1, which are equivalent to residues L84 and M88 of Sc-BUB1_(29-230)_, form part of the corresponding hydrophobic pocket of the Blinkin binding region of Hs-BUB1 and abolish the interaction with Blinkin (Figure 5A), further supporting the idea that this region is important for BUB1 function.

## Discussion

Until now, functions of the various regions of BUB1 have been assigned using deletion mutagenesis, cell localization, immunoprecipitation, and other techniques (Taylor and McKeon, 1997; Vigneron et al., 2004; Larsen et al., 2007). The structure of Sc-BUB1_(29-230)_ provides perspective for examining the functions of BUB1 and its homologs. The structure shows that a divergent triple tandem arrangement of the TPR motif constitutes a suitable structural framework for the physical linkage of BUB1 to interacting partners. The high conservation of the N-terminal region among BUB1 from different species and the use of functional analysis in vivo allow us to define the discontinue Blinkin binding region of Hs-BUB1 and identify two motifs of unique structure features, GN/DD and GIG. These motifs are of particular importance for the interaction of Hs-BUB1 with the N-terminal region of the human mitotic checkpoint factor Blinkin. It would be interesting to explore whether the Hs-BUB1-Blinkin interaction has a role, if any, in directional kinetochore assembly mediated by Hs-Blinkin. This is relevant because Hs-Blinkin also interacts with the kinetochore proteins Zwint-1 and the two subunits hMis13 and hMis14 of the hMis12 complex (Kiyomitsu et al., 2007).

Hs-BUB1 and Hs-BUBR1 both bind Hs-Blinkin through the conserved TPR core of the N-terminal domain (this work; S.D., V.M.B-G., and T.L.B., unpublished data). Hs-Blinkin binds tighter to Hs-BUB1 than Hs-BUBR1, suggesting that additional residues in Hs-Blinkin and/or Hs-BUB1/Hs-BUBR1 participate in the interaction. Alternatively, concerted regulation of Hs-BUB1 and Hs-BUBR1 during mitosis might occur through the Blinkin binding region and/or be modulated depending upon the phosphorylation state of the N-terminal domain of Blinkin. Also, certain residues in Blinkin might dictate the specific interaction with Hs-BUB1 and different ones the interaction with Hs-BUBR1. The stronger and broader affinity of Hs-BUB1 for Blinkin suggests that Hs-BUB1 binds to this protein first, followed by Hs-BUBR1. In any case, the relative affinity of Blinkin for binding Hs-BUB1 and Hs-BUBR1 is likely to be important not only for kinetochore-microtubule attachment, but also for the checkpoint activation functions mediated by these proteins (Figure 6C). The analysis of contacts in the crystal lattice suggests that clusters of highly conserved residues are engaged in Sc-BUB1 dimer formation. However, only the monomeric form of the corresponding N-terminal region of Hs-BUB1 and Hs-BUBR1 have been observed under similar conditions, suggesting that homodimer formation is not a prerequisite for the function of these proteins. Mass spectrometry analysis of cell extracts showed that stringent buffer conditions/washing steps were needed to isolate full-length Hs-BUB1 free from contaminant proteins, including Blinkin and BUBR1 (data not shown). This requirement hampered the determination of whether dimeric full-length Hs-BUB1 was present in cell extracts. Yeast-two hybrid assays have shown the physical interaction between the N-terminal domains of Hs-BUB1 and Hs-BUBR1 is weak (Kiyomitsu et al., 2007). However, the formation of Hs-BUB1-BUBR1 heterodimers could not be detected by analytical gel-filtration chromatography, analytical ultracentrifugation, chemical cross-linking, and native gel electrophoresis, suggesting that the Hs-BUB1-BUBR1 interaction is of transient nature (data not shown).

It is possible that the N-terminal region of BUB1 functions as a docking site for the interaction with other, yet unidentified, BUB1 interacting partners. In this respect, it will be interesting to establish whether this BUB1 domain physically interacts with SGO1, because it has been shown that residues 1–179 of BUB1 from fission yeast are important for targeting SGO1 to centromeres (Vaur et al., 2005). If this turns out to be the case, the interaction would shed light into the role of BUB1 in chromosome alignment and Hs-BUB1-dependent centromere localization of the complex comprising SGO1-MEI-S332 and PP2A phosphatase (Kitajima et al., 2005; Watanabe, 2005).

Two CDC20-binding regions have been identified in Hs-BUBR1 (Davenport et al., 2006; Murray and Marks, 2001). One of these regions (residues 1–477) is highly specific for CDC20 already bound to MAD2 and seems to involve large segments of BUBR1 (Davenport et al., 2006). In budding yeast, the N-terminal region of BUBR1 (MAD3) plays a critical role in CDC20 turnover during mitosis (King et al., 2007). In a similar fashion, the roles of CDC20 and CDH1 in activation are mediated by binding of their extended C-terminal region to the TPR motifs of the APC/C components APC3, APC7, and APC10 (Vodermaier et al., 2003). Interestingly, recent evidence suggests that MAD3 is also a substrate of the APC/C (Burton and Solomon, 2007). Whether the TPR units of MAD3 play a role in the physical interaction with APC/C remains to be established. A further interesting clue about binding sites in BUB1 comes from the observation that there is electron density for a CHES molecule bound to each protomer of the dimer, albeit that bound to chain A in Protein Data Bank 3ESL has weaker density. Each CHES molecule is located in a small hydrophobic pocket formed by residues L62, M65, I66, S69, L84, M88, I110, W113, and L117 involving helices H2, H3, and H4 (Figure 6D). Of these I110, W113 and L117 contribute approximately 50% of the buried area in the CHES-Sc-BUB1_(29-230)_ interface. Examination of the CHES binding site of Sc-BUB1_(29-230)_ shows that L84 is fully conserved across species and M65 and M88 are conservatively varied in BUB1 and BUBR1 from vertebrates (Ile and Leu, respectively). However, if this is a functional site, ligands must vary. The findings that L62, which is conserved in BUB1 and MAD3 from fission and budding yeast, is substituted by a glutamic/aspartic acid residue in higher organisms, that S69 is substituted by a glutamic acid residue, and that W113 is substituted by lysine in BUB1 and BUBR1 from vertebrates, support this notion.

Some of the substitution mutants targeting the TPR core display different phenotypes in Hs-BUB1 and Hs-BUBR1 (Kiyomitsu et al., 2007). Given the association of Hs-BUB1 and Hs-BUBR1 in oncogenesis and aneuploidy (Kops et al., 2005) and the oncogenic property of the fusion of Hs-Blinkin (AF15q14) with the MLL gene in acute myeloid leukemia and lung cancer (Hayette et al., 2000), it would be worthwhile to explore further whether the N-terminal region of Hs-BUB1 (and also that of Hs-BUBR1) constitutes a suitable target for drug discovery. This feature, along with the observation of specific binding of CHES to the hydrophobic pocket of Sc-BUB1_(29-230)_, open up the possibility of an attractive binding site for chemical entities that might be useful tools for characterizing its role and possibly—in the longer term—a target site for drug design. In conclusion, the crystal structure of the conserved N-terminal region of BUB1 provides insight into the recognition mechanism by which the spindle checkpoint protein BUB1 is recruited to kinetochores, and suggests that disease-causing mutations located in the Blinkin binding region operate under a mechanism that is independent of the binding of Hs-BUB1 to Blinkin.

## Experimental Procedures

Experimental procedures are described in the Supplemental Data.

## Figures and Tables

**Table 1:** Data Collection and Refinement Statistics

	Native	Se-Met Crystal
Data Collection		

Space group	C2	C2
Cell dimensions		
*a*, *b*, *c* (Å)	a = 130.30, b = 59.78, c = 71.29	a = 130.86, b = 59.95, c = 71.05
α, β, γ (^o^)	90, 97.96, 90	90, 97.68, 90
		Peak
Wavelength	0.9792	0.9792
Resolution (Å)	50-1.74 (1.78−1.74)	50-1.80 (1.84−1.80)
R_merge_	3.9 (2.49)	7.2 (18.7)
*I* / σ*I*	18.6	10.6
Completeness (%)	98.2 (92.8)	99.9 (100)
Redundancy	4.1 (3.5)	7.0 (6.9)

Refinement		

Resolution (Å)	34.92-1.74	
No. reflections	52,138	
R_work_ / R_free_	18.9/21.6	
No. atoms		
Protein	3465	
CHES molecules	26	
Water	234	
Average *B*-factors (Å^2^)	30.4	
Rms deviations		
Bond lengths (Å)	0.009	
Bond angles (°)	1.075	

One crystal was used for each data set. The statistics shown in parentheses are for the highest-resolution shell.

**Table 2:** Mutations in the N-Terminal Region of Hs-BUB1 Associated with Cancer

Residue *Hs-BUB1*	Amino Acid Substitution	Clinical Condition	Reference	Localization in the Structure	Predicted Effect (I-Mutant method, pseudo-ΔΔG, Kcal/mol)
36	E→D	Colorectal cancer	(Cahill et al., 1998)	H2 (TPR1)	Destabilizing (−0.04)
76–141	Frameshift	Colorectal cancer	(Cahill et al., 1998)	Deletion of H5 (TPR2), H6 and H7 (TPR3)	Destabilizing
130	A→S	Lymph node metastasis	(Shichiri et al., 2002)	Short-loop region that connects helix H7 (TPR3) with “the capping helix” (H8)	Destabilizing (−0.94)
140	Transition of the splicing donor site	Colorectal cancer	(Cahill et al., 1998)	Unknown	
151	H→D	Lung cancer	(Gemma et al., 2000)	Loop region immediately downstream the “capping helix” (H8)	Destabilizing (−0.52)

## Accession Numbers

The coordinates of the N-terminal domain of Sc-BUB1 have been deposited in the Protein Data Bank under the accession code 3ESL.

## Supplemental Data

Document S1. Two Figures, Supplemental Experimental Procedures, and Supplemental References
