# Supplementary material for: The Crystal Structure of the N-Terminal Region of BUB1 Provides Insight into the Mechanism of BUB1 Recruitment to Kinetochores
Source: Structure. 2009 Jan 14;17(1):105–16. doi: 10.1016/j.str.2008.10.015 (PMC2683949; doi:10.1016/j.str.2008.10.015)
Supplement: Document S1. Two Figures, Supplemental Experimental Procedures, and Supplemental References [file mmc1.pdf]

Supplemental Data

The Crystal Structure of the N-Terminal Region of BUB1 Provides Insight into the Mechanism of BUB1 Recruitment to Kinetochores

Victor M. Bolanos-Garcia, Tomomi Kiyomitsu, Sheena D'Arcy, Dimitri Y. Chirgadze, J. Günter Grossmann, Dijana Matak-Vinkovic, Ashok R. Venkitaraman, Mitsuhiro Yanagida, Carol V. Robinson, and Tom L. Blundell

SUPPLEMENTAL EXPERIMENTAL PROCEDURES

Expression and Purification

Recombinant yeast BUB1 N-terminal region residues 29-230 (Sc-BUB1$_{(29-230)}$) fused to an N-terminal GST tag was expressed in *E. coli* BL21(DE3) cells according to reported protocols (Bolanos-Garcia et al., 2005). The expression protocol was modified for incorporation of L-selenomethionine (SeMet) into the protein as follows. A seed culture was prepared in LB medium (20 mL) and grew overnight at 37°C. 1 mL of this culture was used to inoculate 250 mL of M9 broth prepared in 2 L baffled flasks that were previously equilibrated at the same temperature. M9 medium was supplemented with 1 mL of 4.2 g/L $Fe_2(SO)_4$ (sterile filtered), 1 mL of $MgSO_4$ at 1M concentration (autoclaved), 10 mL of glucose at 40% (w/v) (sterile filtered) and 100 µL of 0.5% (w/v) thiamine (sterile filtered) per litre of culture. Growth was continued until absorbance at 600 nm was 0.3. At this point, a solution of L-Lysine, L-Phenylalanine and L-Threonine (all at 100 mg/L) as well as L-Isoleucine, L-Leucine, L-Valine and L-Selenomethionine (all at 50 mg/L) was added to the cultures. After 15 min for inhibition of Met synthesis to start, protein expression was induced with 1 mM IPTG. After 6 hr of growth, cell were harvested, flash-frozen in liquid nitrogen and stored at -80°C.

Mapping Sequence Conservation

To map the sequence-conservation onto the crystal structure of Sc-BUB1$_{(29-230)}$), the BUB1 sequence from *Saccharomyces cerevisiae* with those of homologs from *Schizosaccharomyces pombe*, *Drosophila melanogaster*, *Xenopus laevis*, *Mus musculus* and *Homo sapiens* as well as BUBR1 from the same organisms were input into ClustalW (Thompson et al., 1994). The aligned sequences in multiple formats were converted into a property file by ProSkin (http://www.mcgnmr.ca/ProtSkin/) (Deprez et al., 2005). The property file was then visualized using GRASP (Nicholls et al., 1991).

Crystallization, Data Collection, and Structure Solution

Sc-BUB1$_{(29-230)}$ was crystallized by the hanging drop vapour diffusion method. Native and SeMet-incorporated protein (3 µL) was mixed with 3 µL of mother liquor using a sodium/potassium tartrate condition derived from sparse matrix screening. Before data collection, all crystals were stabilized by gradual transfer to cryoprotectant solutions of the mother liquor. The final cryoprotectant solution contained 35% (v/v) glycerol. Crystals were flash frozen in liquid nitrogen. All X-ray data collection experiments were performed at 100K temperature at the European Synchrotron Radiation Facility (Grenoble France) beamline ID14.4 using x rays of energy of

12,661 eV Wild-type crystals diffracted to 1.74 Å, and selenomethionine-substituted crystals diffracted to 1.80 Å. Both wild-type and selenomethionine crystals belonged to space group C2 and contained two molecules of BUB1 in the asymmetric unit resulting in approximately 56% solvent content. All diffraction data were indexed, scaled and merged using HKL suite (Otwinowski and Minor, 1997).
The initial sets of phases for BUB1 structure were obtained using a single anomalous diffraction technique using data from the selenomethionine-substituted crystals. The electron density maps prior to model building were subjected to a variety of density modification techniques including solvent flattening. Seleno sites were found and refined using PHENIX (Python-based Hierarchical ENvironment for Integrated Xtallography) (Adams *et al*, 2002). Refinement with REFMAC5 (Murshudov et al., 1997) produced high quality $2F_o - F_c$ and $F_o - F_c$ electron density maps. The model was improved by rounds of refinement with REFMAC5 and manual rebuilding using COOT (Emsley and Cowtan, 2004) to an R factor of 18.9% and R_{free} = 21.6% for all reflections in the test set with Bragg spacing between 50 and 1.74 Å. (Table I). The unbiased SAD-phased electron density maps were used to prevent model bias throughout refinement. Electron density maps obtained from these initial phases and native amplitudes clearly revealed all α-helices and most of the connecting loops and were readily traced. Ramachandran plot analysis using the programme RAMPAGE (Lovell et al., 2003) showed that all residues fall in the most favoured Ramachandran category, whilst PROCHECK (Laskowski et al., 1993) shows that 95.2% residues fall in the most favoured Ramachandran category and 4.8.0% in the allowed category.

Protein Analysis
Limited proteolysis, MALDI-TOF, amino acid composition analysis and N-terminal sequencing by the Edman degradation method were conducted at the PNAC facility (Department of Biochemistry, University of Cambridge).

Mass Spectrometry
Samples for nano-electrospray (nano-ES) mass spectrometry were prepared by dilution of the original 220 μM stock solution to a final concentration of 45 μM and buffer exchanged to 0.2 M ammonium acetate at pH 9 using Micro biospin chromatography columns. Mass spectra were recorded on a modified Q-TOF-2 (Sobott et al., 2002). Nano-ES capillaries were prepared in-house from borosilicate glass tubes of 1 mm outer diameter and 0.50 mm inner diameter (Harvard Apparatus, Kent) using a Flaming/Brown P-97 micropipette puller (Sutter Instrument Company, Intracel, Hertfordshire, UK), and gold coated using an S. E. sputter coater (Polaron, Energy Beam Sciences, MA). The capillary tips were cut under a stereo microscope to give inner diameters of 1-5 μm, and 2 μl of protein solution was loaded for sampling. The pressures and accelerating potentials in the mass spectrometer were optimized to remove adducts while preserving non-covalent interactions. The optimum experimental conditions were obtained with a cone voltage of 135 V and capillary voltage of 1.5 kV. External calibration of the spectra was achieved using solutions of caesium iodide, and the errors from calculated values for all reported masses are approximately 0.1%. Data acquisition and processing were performed using the MassLynx software (Micromass).

Small-Angle X-ray Scattering
Solution x-ray scattering data were collected at station 2.1, Synchrotron Radiation Source, Daresbury Laboratory UK, using a two-dimensional multiwire proportional counter at sample-to-detector distances of 1m and 4.25m and an x-ray wavelength of 1.54 Å with beam currents between 120 and 200 mA. Each sample was exposed for 25 min in 30 s frames. Frames at the beginning and the end of each data collection were compared to exclude the possibility of protein aggregation and/or radiation

damage. The data reduction involved radial integration, normalization of the one-dimensional data to the intensity of the transmitted beam, correction for detector artefacts, and subtraction of buffer scattering (OTOKO, SRS Daresbury). The q-range was calibrated with an oriented specimen of wet rat tail collagen (diffraction spacing 670 Å) and silver behenate (diffraction spacing 58.38 Å). Sc-BUB1$_{(1-230)}$ and Sc-BUB1$_{(29-230)}$ solutions at concentration ranging between 1 and 15 mg/ml (≈ 36-550 μM) were prepared in 0.05 M Tris-HCl, 0.15 M NaCl, pH 8.0 and analysed at 20°C. The profiles collected at both camera lengths were merged so as to cover the momentum transfer interval 0.012 Å^{-1} < q < 0.778 Å^{-1}. The modulus of the momentum transfer is defined as $q = 4 \pi \sin \Theta/\lambda$, where 2Θ is the scattering angle and λ is the wavelength used. The maximum scattering angle corresponds to a nominal Bragg resolution of approximately 8 Å. The forward scattering intensity, radius of gyration R_g, and the maximum particle dimension D_{max}, and intraparticle distance distribution function [p(r)] were calculated from the scattering data using the indirect Fourier transform method program GNOM (Svergun, 1991). X-ray scattering predicted on the basis of the crystal structure of Sc-BUB1$_{(29-230)}$ was compared to its conformation in solution using the program CRYSOL (Svergun et al., 1995) which simulates the scattering profile from atomic coordinates and provides a goodness-of-fit relating to the experimental data by inclusion of a hydration shell. For determinations of protein oligomerisation states, the following proteins were used as reference: BSA (67 kDa); Ovalbumin (43 kDa); Thaumatin I (22 kDa).

Circular Dichroism

Far-UV circular dichroism spectra were recorded on an AVIV 62-S spectropolarimeter (AVIV, New Jersey, USA) previously calibrated with camphorosulfonic acid and equipped with a temperature control unit. In all experiments, spectra were recorded at 20°C in a 0.1 cm quartz cell using an average time of 1.0 s, a step size of 0.5 nm, 1-nm bandwidth, and averaged over 20 scans. The dependence of CD signal on protein concentration was calculated by triplicate using independent samples of concentrations ranging between 50 μg/ml to 600 μg/ml (i.e., ≈ 2-22 μM). After subtraction of the buffer baseline, the CD data were normalized and reported as molar residue ellipticity. For studies on protein stability as a function of pH, protein solutions were prepared in 0.05 M sodium acetate, pH 4 and 5; 0.05 M sodium phosphate buffer, pH 6, 7, 8 and 0.05 M sodium borate, pH 9 and 10. For thermal denaturation experiments, five unfolding curves were recorded upon heating from 5 to 95°C at a rate of 1° C/min, and 80 s accumulation time. The apparent melting temperature, Tm, was determined from differential melting curves of the function $d[\theta_{222}](T)/dt$. The concentration of protein solutions was determined from amino acid composition analysis at the PNAC facility (Department of Biochemistry, University of Cambridge). Far-UV CD analysis of all proteins was carried out immediately after gel filtration chromatography.

Optimal Docking Area Analysis

The ODA method identifies protein surface patches with propensity for protein-protein interactions on the basis of desolvation energy (Fernandez-Recio *et al*, 2005). The method calculates the effective desolvation energy for burying a continuous surface patch from the solvent according to atomic solvation parameters (Fernandez-Recio *et al*, 2004). The docking surface energy is calculated for patch residues that lie within a sphere of radius d (where d = 1, 2, . . ., 20 Å) from the centre of coordinates of each residue side chain. The patch with lowest desolvation energy value is called an ODA. Significant ODAs are those with an optimal desolvation energy of less than -10.0 kcal/mol, and can be graphically displayed on the surface of the proteins by colouring each surface residue according to its ODA value (i.e., from red to blue indicating increasing propensities for a docking site). The ability of the ODA method

to identify protein interaction sites has been validated on a set of three-dimensional structures of non-homologous proteins involved in non-obligate protein–protein interactions. Significant ODAs were found in half of the test proteins, and >80% of the ODAs identified corresponded to a known protein interaction site (Fernandez-Recio *et al*, 2005).

Yeast 2-Hybrid Analysis

The 2-hybrid analysis was performed according to the guidelines described by the manufacturer (MATCHMAKER 2-hybrid analysis kit, Clontech) using pGBT9 and pGAD424. The β-galactosidase filter assay was performed using the SFY526 strain that carried the GAL1-lacZ reporter according to conventional methods.

SUPPLEMENTAL REFERENCES

Adams, P.D., Grosse-Kunstleve, R.W., Hung, L.-W., Ioerger, T.R., McCoy, A.J., Moriarty, N.W., Read, R.J., Sacchettini, J.C., Sauter, N.K., and Terwilliger, T.C. (2002). PHENIX: building new software for automated crystallographic structure determination Acta Cryst. *D58*, 1948-1954.

Emsley, P., and Cowtan, K. (2004). Coot: model-building tools for molecular graphics. Acta Crystallogr. D Biol. Crystallogr. *60*, 2126-2132.

Fernandez-Recio, J., Totrov, M., Skorodumov, C., and Abagyan, R. (2005). Optimal docking area: a new method for predicting protein-protein interaction sites. Proteins *58,* 134-143.

Laskowski, R.A., MacArthur, M.W., Moss, D.S., and Thornton, J.M. (1993). PROCHECK: a program to check the stereochemical quality of protein structures. J. Appl. Cryst. *26*, 283-291.

Lovell, S.C., Davis, I.W., Arendall, W.B. 3$^{rd.}$, de Bakker, P.I., Word, J.M., Prisant, M.G., Richardson, J.S., and Richardson, D.C. (2003). Structure validation by Calpha geometry: phi,psi and Cbeta deviation. Proteins *50*, 437-450.

Murshudov, G.N., Vagin, A.A., and Dodson, E.J. (1997). Refinement of Macromolecular Structures by the Maximum-Likelihood Method. Acta Cryst. D Biol. Crystallogr. *53*, 240-255.

Otwinowski, Z., and Minor, W. (1997). Processing of X-ray Diffraction Data Collected in Oscillation Mode. Methods Enzymol. *276*, 307-326.

Sobott, F., Hernandez, H., McCammon, M. G., Tito, M. A., and Robinson, C. V. (2002). A tandem mass spectrometer for improved transmission and analysis of large macromolecular assemblies. Anal. Chem. *74*, 1402-1407.

Svergun, D.I. (1991). Mathematical methods in small-angle scattering data analysis. J. Appl. Crystallogr. *24,* 485-492.

Svergun, D., Barberato, C., and Koch, M.H.J. (1995). CRYSOL - A program to evaluate X-ray solution scattering of biological macromolecules from atomic coordinates. J.Appl.Cryst. *28*, 768-773.

Thompson, J.D., Higgins, D.G., and Gibson, T.J. (1994). CLUSTAL W: improving the sensitivity of progressive multiple sequence alignment through sequence weighting, position-specific gap penalties and weight matrix choice. Nucleic Acids Res. *22*, 4673-4680.

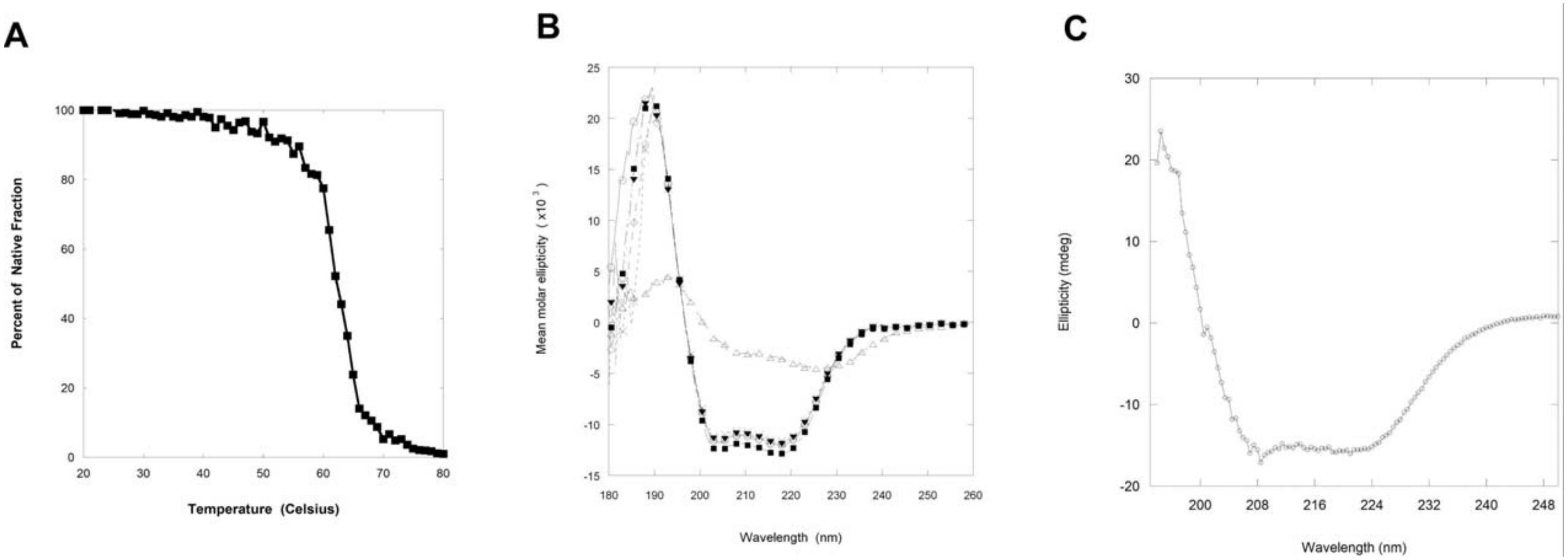

Figure S1. Far-UV CD Spectroscopy of Sc-BUB1$_{(29\text{-}230)}$

(A) Thermal denaturation profile monitored at 222 nm.

(B) Stability of this domain monitored at pH 4 (Δ), 6 (○), 7 (■), 8 (◊), 9 (×) and 10 (▲).

(C) The Sc-BUB1$_{(29\text{-}230)}$ mutant G146A-G148A shows a similar secondary structure as the native protein, suggesting its adopts a native-like fold.

In all studies protein concentration was 600 µg/ml (i.e., ≈ 24 µM).

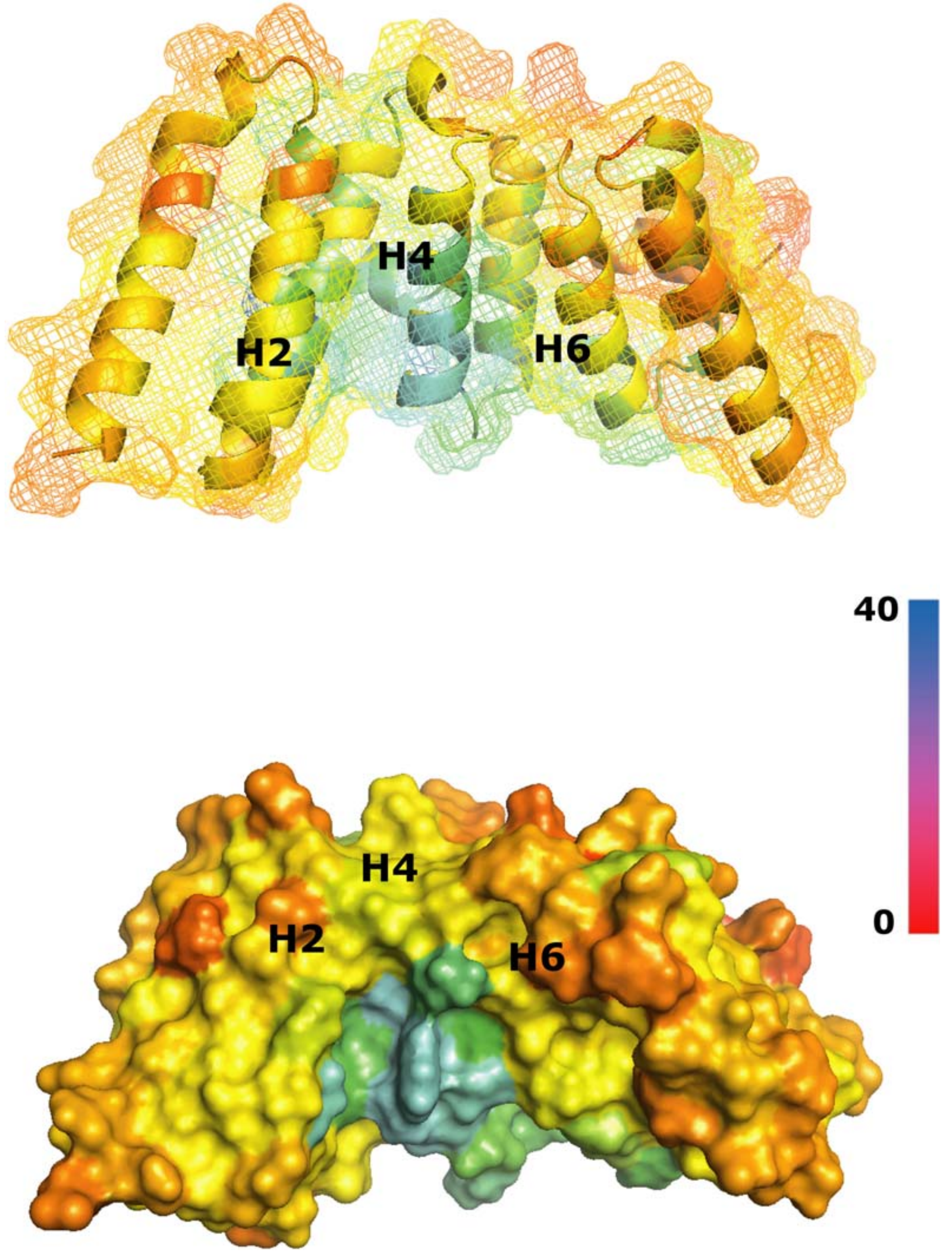

Figure S2. Analysis of Putative Protein-Protein Interaction Sites
Optimal Docking Area (ODA) of Sc-BUB1$_{(29\text{-}230)}$. The absolute ODA scale, 0-40, is shown on the right upper corner; the colours, ranging from red to blue, represent increasing propensities for a binding site. The projection of ODA onto the protein surface shows that the region of highest propensity for protein-protein interactions involves α-helices H2, H4 and H6.
